# A Chemical Activity Approach to Exposure and Risk Assessment of Chemicals

**DOI:** 10.1002/etc.4091

**Published:** 2018-05

**Authors:** Frank A.P.C. Gobas, Philipp Mayer, Thomas F. Parkerton, Robert M. Burgess, Dik van de Meent, Todd Gouin

**Affiliations:** a Resource and Environmental Management, Simon Fraser University, Burnaby, British Columbia, Canada; b DTU Environment, Department of Environmental Engineering, Technical University of Denmark, Lyngby, Denmark; c Toxicology & Environmental Science Division, ExxonMobil Biomedical Sciences, Houston, Texas, USA; d US Environmental Protection Agency, ORD/NHEERL, Atlantic Ecology Division, Narragansett, Rhode Island; e Department of Environmental Science, Radboud University Nijmegen, Nijmegen, The Netherlands; f TG Environmental Research, Sharnbrook, Bedfordshire, United Kingdom

**Keywords:** Risk assessment, Environmental fate, Hazard/risk assessment, Mixture, Bioaccumulation, Exposure

## Abstract

To support the goals articulated in the vision for exposure and risk assessment in the twenty-first century, we highlight the application of a thermodynamic chemical activity approach for the exposure and risk assessment of chemicals in the environment. The present article describes the chemical activity approach, its strengths and limitations, and provides examples of how this concept may be applied to the management of single chemicals and chemical mixtures. The examples demonstrate that the chemical activity approach provides a useful framework for 1) compiling and evaluating exposure and toxicity information obtained from many different sources, 2) expressing the toxicity of single and multiple chemicals, 3) conducting hazard and risk assessments of single and multiple chemicals, 4) identifying environmental exposure pathways, and 5) reducing error and characterizing uncertainty in risk assessment. The article further illustrates that the chemical activity approach can support an adaptive management strategy for environmental stewardship of chemicals where “safe” chemical activities are established based on toxicological studies and presented as guidelines for environmental quality in various environmental media that can be monitored by passive sampling and other techniques.

## INTRODUCTION

Since Rachel Carson’s (1962) landmark book *Silent Spring* generated global awareness of the potential harm from large-scale chemical use, hazard, exposure, and risk assessment of chemicals have become an integral part of national and international efforts to regulate chemical use. The general goal of such assessments is to contribute information for managing chemicals that benefit society while safeguarding the environment and human health from detrimental and potentially irreversible harm. A common assessment strategy involves evaluating the persistence, bioaccumulation, toxicity, and long-range transport potential of chemicals (United Nations Environment Programme 2001). This approach, which does not involve actual exposure and risk assessment, is often used to screen and prioritize chemicals for further assessment and/or control substances (US Environmental Protection Agency 1976; Government of Canada 1999; European Commission 2006; Japanese Ministry of the Environment 2011). Environmental risk assessment of chemicals often includes the use of risk quotients, where actual or predicted concentrations of the chemical in a particular environmental medium are compared with toxicity measures (e.g., no-observed-adverse-effect concentrations) by using concentration ratios. One example of this approach is the ratio of the predicted-environmental concentration to the predicted-no-effect concentration (Wilkinson et al. 2000). Methods used to assess hazards of chemicals to multiple species include species sensitivity distributions, which when combined with exposure information can be used to estimate ecological risks that are often expressed as the fraction of potentially affected species (Suter 1993). Methods for the assessment of risks of chemicals to human populations include the calculation of hazard assessment indices and excess lifetime cancer risks (US Environmental Protection Agency 1984). In most cases, hazard screening, prioritization, and subsequent risk assessments are performed on a chemical-by-chemical basis. Risk assessment of mixtures of chemicals is often addressed by direct toxicity assessment (e.g., whole-effluent toxicity tests) or the application of models that account for the mode of action of the chemical (European Commission 2012).

The National Research Council has published a series of reports outlining a strategy for conducting exposure science and toxicity testing in the twenty-first century (National Research Council 2007, 2012). The reports promote the creation of large databases of information, particularly related to developing an improved mechanistic understanding of toxicity pathways and new tools for toxicity testing. These initiatives include an adverse outcome pathway approach to organize and communicate mechanistic toxicity pathways from molecular initiating events to adverse effects at the individual, population, and community levels (Ankley et al. 2009) and computational methods that examine chemical structures to screen against a variety of hazard endpoints and bioinformatics. They also include various rapid high-throughput in vitro assays, notably the US Environmental Protection Agency’s (USEPA’s) ToxCast program (Dix et al. 2007). These methods make it possible to screen thousands of chemicals for toxicity in a period of one to several years. The vision also includes the collection, compilation, and evaluation of a range of exposure data for human and wildlife target species generated by various methods, including (bio)sensors, passive sampling, market data, remote sensing, and geographical information technologies (National Research Council 2007). Databases, such as the USEPA’s ExpoCast, are currently being developed to compile exposure data for advancing exposure characterization (Cohen et al. 2010).

The vision for the twenty-first century also recognizes some key challenges (Krewski et al. 2014). One challenge is the development of methods that can adequately interpret the vast growing body of exposure and toxicity information and the uncertainty associated with the data obtained from various sources. A second challenge is the risk characterization of chemical mixtures. Addressing these challenges will help translate the value of toxicological and exposure knowledge into chemical-management decisions. The objective of the present Focus article is to address these challenges by highlighting the potential application of a thermodynamic-based framework that relies on the concept of chemical activity for expressing and interpreting exposure and toxicity information in support of robust, quantitative risk assessments and corresponding management actions.

Chemical activity and a related quantity, fugacity, were introduced by Lewis (1901, 1907). They express a chemical’s potential for distribution and reaction in the environment. Chemical activity and fugacity are widely used in the field of chemical engineering to describe the distribution of chemicals between different phases and media. In the field of medicine, the application of chemical activity has been instrumental in predicting the action of anesthetic drugs used in surgery (Overton 1896; Meyer 1899). In toxicology and ecotoxicology, fugacity and chemical activity approaches have been applied to interpret and study both the uptake (Connolly and Pedersen 1988; Gobas et al. 1999; Nfon and Cousins 2007; Burkhard et al. 2012) and the toxicity (Ferguson 1939; Freidig et al. 1999; Schmidt et al. 2013; Armitage et al. 2014; Thomas et al. 2015) of chemicals in organisms. Fugacity and related models (for neutral nonpolar organic chemicals) and related aquivalency models (for metals) are widely used for assessing contaminant flows and distribution in the environment (Di Toro et al. 1991; Mackay 2001; Schwarzenbach et al. 2003; Mackay and Arnot 2011). Fugacity and chemical activity approaches have also been used for regulatory purposes and to conduct chemical risk assessment (Mackay et al. 2011; Gobas et al. 2016, 2015b; Fairbrother and Woodburn 2016). The European Centre for Ecotoxicology and Toxicology of Chemicals (2016) recently published a report on defining the role of chemical activity in environmental risk assessment, discussing both opportunities and challenges to the application of the chemical activity approach to environmental risk assessment.

Because of its fundamental role in environmental distribution, toxicity, and risk assessment, the chemical activity approach may provide a useful unifying framework for the integration of information from exposure monitoring and modeling studies and in vivo and in vitro toxicity tests to elucidate exposure pathways, toxicity, and the magnitude of risk that a substance may pose. In the present article, we illustrate how a chemical activity approach can be used to support chemical management. First, we describe the chemical activity approach and its strengths and limitations. Second, we describe how chemical activity can be determined and estimated. Third, we provide several examples of how the chemical activity approach can inform and support exposure, hazard, and risk assessments. Fourth, we provide suggestions for how the chemical activity approach can be applied in a regulatory context to improve the stewardship of chemicals in commerce.

## THEORY

### What is the chemical activity approach?

**FIGURE 1:**
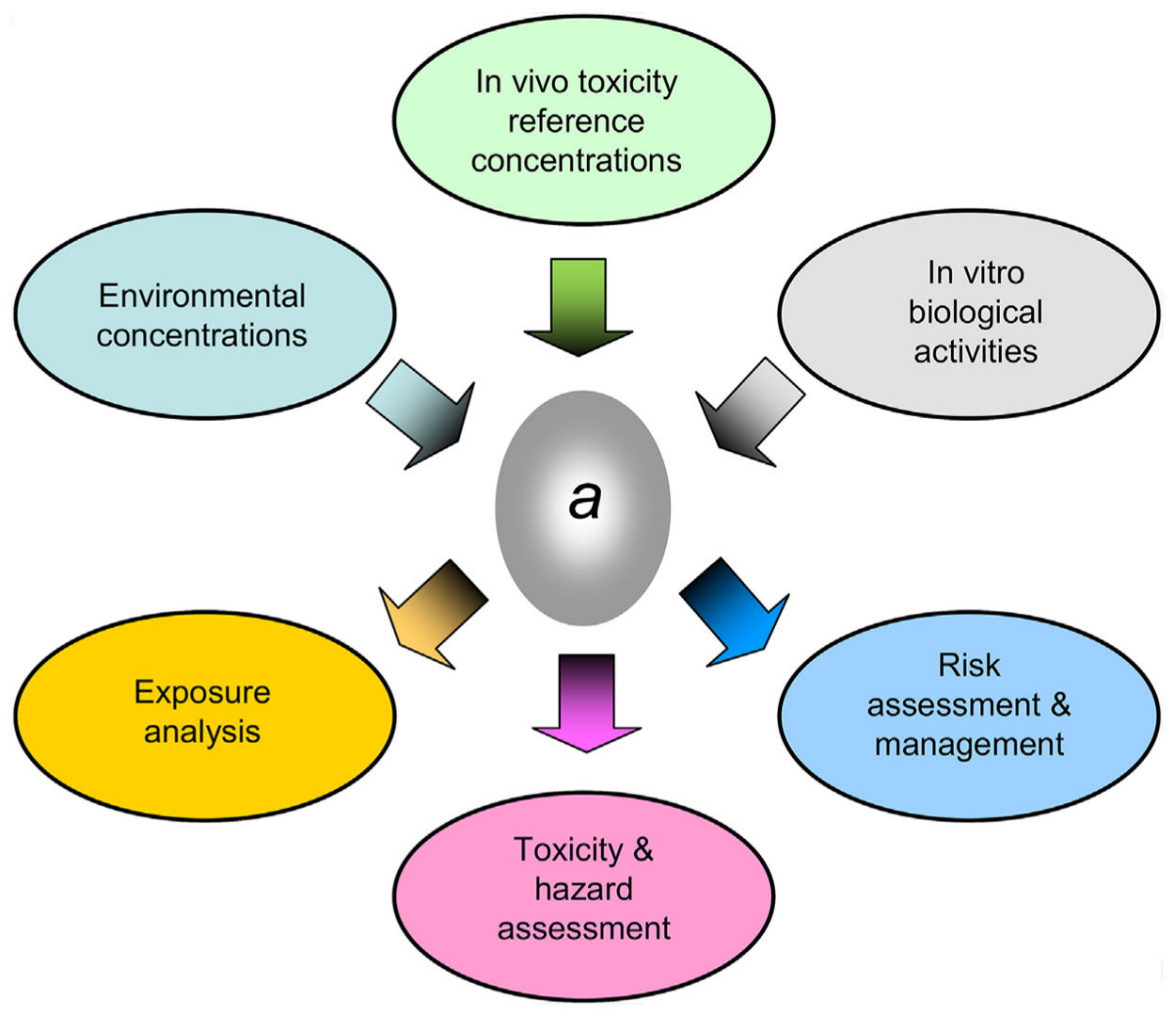
Conceptual diagram illustrating potential applications of the chemical activity approach in environmental exposure and risk assessment. The chemical activity approach involves the expression of exposure concentrations and in vivo and in vitro toxicity reference concentrations in terms of a common unitless metric (i.e., chemical activity) for conducting exposure, hazard, and risk assessment and management. *a =* chemical activity.

As illustrated in Figure 1, the chemical activity approach involves the expression of media-specific concentrations to which organisms are exposed and in vivo and in vitro toxicity concentrations and dosages in terms of a common metric (i.e., chemical activity). Media include ambient water, sediments, soil, air, and fish. Media also include biological fluids such as urine and blood and incubation media used in bioassays. The media can further include materials in passive and active samplers used for environmental monitoring. A range of in vivo and in vitro toxicity metrics such as no-observed-effect concentrations (NOECs), lowest-observed-effect concentrations (LOECs), concentrations causing 50% lethality (LC50s) or 50% nonlethal effects (EC50s), and concentrations causing biological activity in in vitro tests can also be expressed in terms of chemical activities. The various exposure and toxicity measures can then be directly compared because they are all expressed in the same units.

The main strength of the chemical activity approach is its ability to improve and expand the comparison of exposure and biological effect measures in risk assessments. Chemicals in the environment and in experimental systems often occur in multiple, diverse media. The composition of the medium modulates a substance’s distribution between freely dissolved and other constituent phases, thereby influencing the chemical and biological activity of the substance. A comparison of the concentrations of a chemical in different media is often difficult and subject to error because it is tantamount to comparing apples and oranges. Environmental risk assessments of chemicals therefore often rely on a comparison of exposure and toxicity data for a single medium, thereby excluding other available information. For example, a risk assessment may involve the comparison of the concentration of a chemical in ambient water with the concentration of the chemical that produces a biological response in an aquatic toxicity test (e.g., LC50 or EC50). Even in such risk assessments, there may be substantial differences in the composition of the aqueous medium among toxicity tests and ambient water types. But more importantly, many other sources of information on exposure (e.g., concentration data from [bio]sensors and passive sampling devices and concentration data for the chemical in media other than water, such as sludge, sediments, and commercial products) and toxicity (e.g., in vitro response studies) are typically not considered in environmental risk assessment because they cannot be easily compared to aqueous concentrations. This reduces opportunities for gaining knowledge, improving risk characterization, and assessing uncertainty. One of the contributions of the chemical activity approach to exposure and risk assessment is that it provides a method for using concentration data from a variety of environmental media, test systems, and sensor materials in a risk assessment. Examples of such risk assessments are discussed below.

Another contribution of the chemical activity approach is the ability to express the relative potency (or inherent toxicity) of individual and mixtures of chemicals. Several authors have shown that many individual chemicals exert a baseline toxicity that is closely related to the chemical activity of the chemical in organisms. For example, several authors have shown that many individual neutral organic chemicals cause lethality in organisms when they reach chemical activities between 0.01 and 0.1 (Reichenberg and Mayer 2006; Schmidt et al. 2013; Thomas et al. 2015). Mixtures of these chemicals appear to cause the same biological response as the individual chemicals when the combined chemical activity in the organism, determined as the sum of the chemical activities of the mixture’s components, reaches critical values similar to those of the individual components of the mixture (Schmidt et al. 2013; Smith et al. 2013). Chemicals that exert this behavior are believed to act in a nonspecific fashion through dissolution in cell membranes and disrupting critical membrane transfer functions (Franks and Lieb 1982; Kipka and Di Toro 2009). The chemical activity approach provides a relatively simple framework to differentiate between chemicals that cause this type of baseline toxicity and chemicals with a potency greater than that of baseline toxicants, which cause acute lethality at chemical activities in organisms less than 0.01.

**FIGURE 2:**
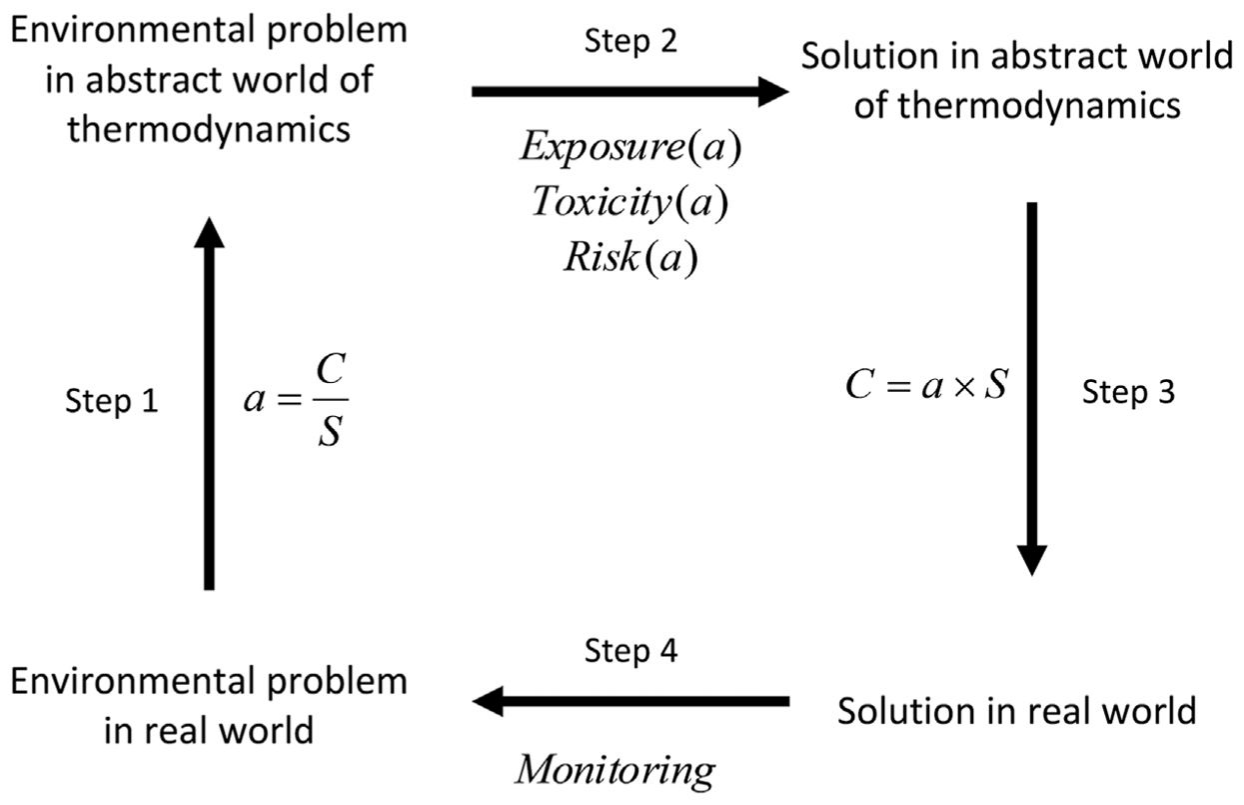
Conceptual diagram illustrating the application of chemical activity to addressing environmental problems. Step 1 involves the conversion of environmental concentrations and toxicity data to chemical activities based on solubility. Step 2 involves the evaluation of exposure and toxicity data expressed in terms of chemical activities. Step 3 involves the conversion of exposure, toxicity, and risk information expressed in chemical activities in terms of concentration units. Step 4 involves the monitoring of exposure, toxicity, and risk to measure the effectiveness of the solution to the environmental problem. *a* = chemical activity; *C* = concentration; *S* = solubility.

A notable weakness of the chemical activity approach is that despite its sound theoretical basis, chemical activity remains an unfamiliar concept beyond the circles of chemists, chemical engineers, and pharmaceutical scientists. Concentration is a much more familiar concept than chemical activity. We therefore envision 2 strategies for the practical application of the chemical activity approach. The first strategy involves the translation of concentrations into chemical activities and vice versa (Figure 2), thereby taking advantage of both the concentration and the chemical activity concepts. Various real-world environmental pollution problems (e.g., what is the combined toxicity of a chemical mixture? What is a safe chemical concentration in water [e.g., water quality guideline]? Does a chemical pose a risk to the environment? Does a chemical biomagnify in a food web?) can be addressed by expressing available concentration data in terms of chemical activities (step 1). The problem can then be framed, evaluated, and possibly solved using the laws and constraints of thermodynamics (step 2). Finally, a solution expressed in thermodynamic quantities can be re-expressed in terms of real-world quantities such as concentrations, dosages, and emission rates (step 3), which can be controlled and/or monitored (step 4). A second strategy for overcoming difficulties with the unfamiliarity of the chemical activity concept is the use of proxies for chemical activity. Possible candidates for such proxies include the freely dissolved chemical concentration in the water (*C*_free_) and the lipid-normalized concentration or equilibrium partitioning concentration in lipids (*C*_lipid_; Webster et al. 1999). Chemical activity can also be viewed as the fraction of chemical saturation. For example, a liquid chemical with a solubility of 1000 μg/L in an environmental medium (e.g., water), will exhibit a chemical activity of 0.01 or 1% at a freely dissolved concentration of 10 μg/L in that medium. Various authors have taken advantage of these approaches to conduct thermodynamically sound analyses of chemical distribution and toxicity behaviors (Connolly and Pedersen 1988; Di Toro et al. 1991; Gobas et al. 1999; Mackintosh et al. 2004; Burkhard et al. 2012; Mayer et al. 2014; Burgess et al. 2015).

### What is chemical activity?

Chemical activity (*a*) is a unitless thermodynamic quantity defined by Lewis (1901, 1907) to describe nonideal solutions of chemicals in different phases and media. Chemical activity is defined in 2 ways. First, it is defined as the ratio of the chemical’s fugacity (*f*), with units of pressure, in an environmental medium and the reference fugacity of the pure chemical at a defined standard state (*f*^R^), also with units of pressure. The reference fugacity is often the fugacity of the pure chemical in its actual state (for chemicals that are in the liquid form at the temperature of interest) or the subcooled liquid state (for chemicals that are in the solid form at the temperature of interest) at the system’s temperature. Second, chemical activity is defined on a Raoult’s law basis as the product of the concentration of the chemical *x* (in units of moles of solute per moles of solvent) and the activity coefficient γ (unitless; Prausnitz 1969):

(1) $$a=f/f^{R}=\gamma \times x$$

For neutral hydrophobic organic chemicals, γ in dilute aqueous solutions (such as those often encountered in the ambient environment and toxicity tests) can be determined from the solubility of the substance in water (Prausnitz 1969). For substances that are liquids at the system’s temperature, γ is equal to the reciprocal of the chemical’s solubility *X* in the solvent in units of moles per mole:

(2) γ=1/X

For substances that are solids at the system’s temperature, the activity coefficient γ of neutral hydrophobic organic chemicals in water is

(3) γ=F/X

where *F* is the fugacity ratio (dimensionless), which is a function of the melting point of the chemical *T*_M_ (K) according to

(4) $$\ln F=-(\Delta H_{f}/R)(1/T-1/T_{M})$$

where Δ*H_f_* is the enthalpy of fusion (joules per mole), *R* is the gas constant (8.314 J/mol/K), *T* is temperature, and *T*_M_ (K) is the melting point of the solid chemical (Prausnitz 1969). Applying Walden’s rule, which states that Δ*H_f_*/*T*_M_ is often approximately 56.5 J/mol, a relatively simple method for estimating *F* from the melting point *T*_M_ and the environmental temperature *T* of the chemical can be obtained (Mackay 2001):

(5) $$F=\exp(-6.79\times [T_{M}/T-1])$$

Assuming that γ is constant over the concentration gradient from 0 to *X*, it follows that the chemical activity (*a*) can be approximated by the ratio of the chemical’s concentration *x* (moles per mole) and its solubility *X* (moles per mole) in the medium in which it occurs. Dividing *x* and *X* by the molar volume of the solvent produces a method to express the chemical activity in more conventional units of chemical concentration *C* (moles per cubic meter) and solubility *S* (moles per cubic meter):

(6) a=x/X=C/S(forliquids)anda=x/X=C×(F/S)(forsolids)

The activity coefficient of neutral hydrophobic organic chemicals in phases other than water (γ_P_), can be approximated by the product of the activity coefficient in water (γ_W_) and the dimensionless, mole fraction–based partition coefficient (*K*_PW_) of the chemical between phase (P) and water (W)

(7) $$\gamma_{P}=K_{\text{PW}}\times \gamma_{W}$$

such that the chemical activity in the nonaqueous phase P (*a*_P_) can be calculated from the molar concentration (*x*_P_) in phase P as

(8) $$a_{P}=\gamma_{P}\times x_{P}$$

A useful property of chemical activity is that it describes the natural tendency of chemicals to achieve a thermodynamic equilibrium. A thermodynamic equilibrium is defined (Lewis 1901, 1907; Prausnitz 1969) in terms of the chemical activities in the media involved (e.g., media i and j) being equal:

(9) $$a_{i}=a_{j}$$

Testing for equilibrium has been shown to provide useful insights into the behavior of chemicals in the environment. Also, equilibrium partitioning is a useful strategy for sensing chemicals in environmental media that is applied in passive sampling techniques. Furthermore, applying equilibrium assumptions can be a useful tool in solving contaminant-related management problems. Examples of these applications of equilibrium partitioning are described below.

### How to determine chemical activity?

Equation 6 establishes a relatively simple method for determining the chemical activity in a medium as the ratio of the concentration of the chemical in a medium and the solubility or sorptive capacity (herein referred to as “solubilities”) of the chemical in that medium. The solubility (moles per cubic meter) of a chemical in pure media such as water (*S*_W_), air (*S*_A_), and lipids (*S*_L_) can usually be determined or estimated from the reported aqueous solubility, Henry’s law constant (H), octanol–water partition coefficient (*K*_OW_), and melting point. For neutral organic chemicals that are not miscible with water, a variety of databases and computational methods exist to determine these solubilities for neutral organic chemicals, for example, EpiSuite (US Environmental Protection Agency 2004), SPARC (Archem 2017), ChemSpider (Pence and Williams 2010), Cosmotherm (Klamt 1995), polyparameter linear free energy relationships (Endo and Goss 2014), and others (Brown et al. 2012). For ionic organic substances, similar databases and computational methods are currently being developed. The solubilities of chemicals in heterogeneous media such as sediment, soil, and biological tissues are not as readily accessible. Even the solubilities of chemicals in natural waters, which may contain particulate and dissolved organic matter, salts, and other components, are typically not readily available. For chemicals in heterogeneous environmental media, a combined solubility (*S_T_*) of the chemical in the environmental matrix can be derived from the solubilities of the chemical in the constituent pure media

(10) $$S_{T}=\sum_{j=1}^{m}\phi_{j}\times S_{j}$$

where *ϕ_j_* is the volume fraction of each component *j* of a particular medium consisting of *m* components. Methods to derive chemical activities from concentrations in a range of environmental and experimental media can be found online (Gobas et al. 2015a) The methods are presented in an Excel spreadsheet and aim to facilitate the calculation of chemical activities. Chemical activity can also be measured in the laboratory and field by passive sampling using various polymers such as polydimethylsiloxane, low-density polyethylene, and polyoxymethylene (Ghosh et al. 2014) as discussed in further detail in the next section.

### Application of chemical activity to exposure assessment

The exposure of chemicals to organisms in the environment is usually measured or calculated in terms of the concentration of the chemical in the medium to which the organism is exposed. However, because environmental media (e.g., water, sediment, diet, air) vary enormously in composition, concentrations of a chemical in different exposure media are difficult to compare and do not provide direct information on the direction of diffusive flux or the bioavailability of the chemical. Applying the chemical activity approach to passive sampler data can address some of the limitations of conventional concentration measurements and improve exposure assessment. Passive samplers are, in most cases, well-defined polymers(e.g., low-density polyethylene, polyoxymethylene, polydimethylsiloxane) that are deployed in environmental media to “sense” contaminant concentrations (Ghosh et al. 2014). Supplemental Data, Table S1, summarizes reported studies applying passive samplers for measuring multimedia chemical activity gradients (e.g., air–water, sediment interstitial water–water column, sediment interstitial water–water column–biotalipid) for a range of organic substances at various locations around the world. Passive samplers can be divided into 2 groups (i.e., equilibrium and nonequilibrium samplers). Equilibrium samplers exhibit relatively fast uptake and depuration kinetics so that substances achieve a chemical equilibrium between the sampler and the medium that is being sampled (Mayer et al. 2003). Equilibrium allows the chemical activity of the chemical in the sampler to be equated to that of the chemical in the medium that is sampled (Legind et al. 2007). Nonequilibrium samplers exhibit slower kinetics and do not achieve a chemical equilibrium between the sampler and the medium that is being sampled. Performance reference compounds can be incorporated into the polymer of the passive samplers so that measured concentrations can be corrected to reflect equilibrium concentrations even when sampler deployments are too brief to achieve equilibrium (Ghosh et al. 2014).

Although the environmental media (e.g., sediment, soil, biological tissues) that are investigated are often complex, variable, and heterogeneous, passive samplers are inert, well-defined materials with a consistent composition. The sorptive capacity or solubility of the chemical in the passive sampler can therefore be measured in a reliable and reproducible fashion (Mayer et al. 2003). In many cases, the solubility of a chemical in the sampling matrix can be more reliably determined than the solubility of the chemical in the environmental medium that is being sensed (Legind et al. 2007). Concentrations of chemicals in passive samplers (*C*_PS_, moles per cubic meter) can therefore readily be expressed in terms of chemical activities (*a*_PS_) as long as the solubilities (*S*_PS_, moles per cubic meter) or partitioning properties (represented by the passive sampler–water partition coefficient *K*_PS_ [unitless] and the subcooled liquid solubility [*S_W_*, moles per cubic meter] in pure water) of the chemical in the sampler matrix are known:

(11) $$a_{\text{PS}}=\frac{C_{\text{PS}}}{S_{\text{PS}}}=\frac{C_{\text{PS}}}{K_{\text{PS}}\times S_{W}}$$

A passive sampler that is deployed in a nondepletive manner (i.e., the passive sampler does not significantly change the mass of the chemical in the medium being sampled) and achieves chemical equilibrium with the medium being sampled (i.e., the chemical activities in the sampler and environmental medium are the same) can therefore determine the chemical activity in the medium in which the sampler is deployed (Ghosh et al. 2014; Burgess et al. 2015). Because the solubility of the chemical in the passive sampler polymer is independent of the matrix in which it is deployed, passive sampling measurements in different environmental phases (e.g., water and sediment organic carbon, or water and fish lipids) can produce chemical activity ratios that reveal the magnitude and direction of diffusive gradients, which in turn indicate directions for net passive transport of the chemical in the environment (Jahnke et al. 2014a, 2014b). For example, Jahnke et al. (2014a, 2014b) used equilibrium sampling of polychlorinated biphenyls (PCBs) within sediment and eel tissue to measure chemical activity ratios between eel and sediment. The chemical activity ratios indicated lower PCB activities in eels compared with sediments, whereas biota to sediment accumulation factors (BSAFs) indicated lipid-normalized concentrations in eels exceeded organic carbon–normalized sediment concentrations (Jahnke et al. 2014b). That study is an example of how equilibrium sampling and chemical activity can facilitate a thermodynamic assessment of bioaccumulation and other environmental fate processes. Such studies also highlight the subtle but sometimes important differences between concentration ratios (e.g., BSAF) and chemical activity ratios.

**FIGURE 3:**
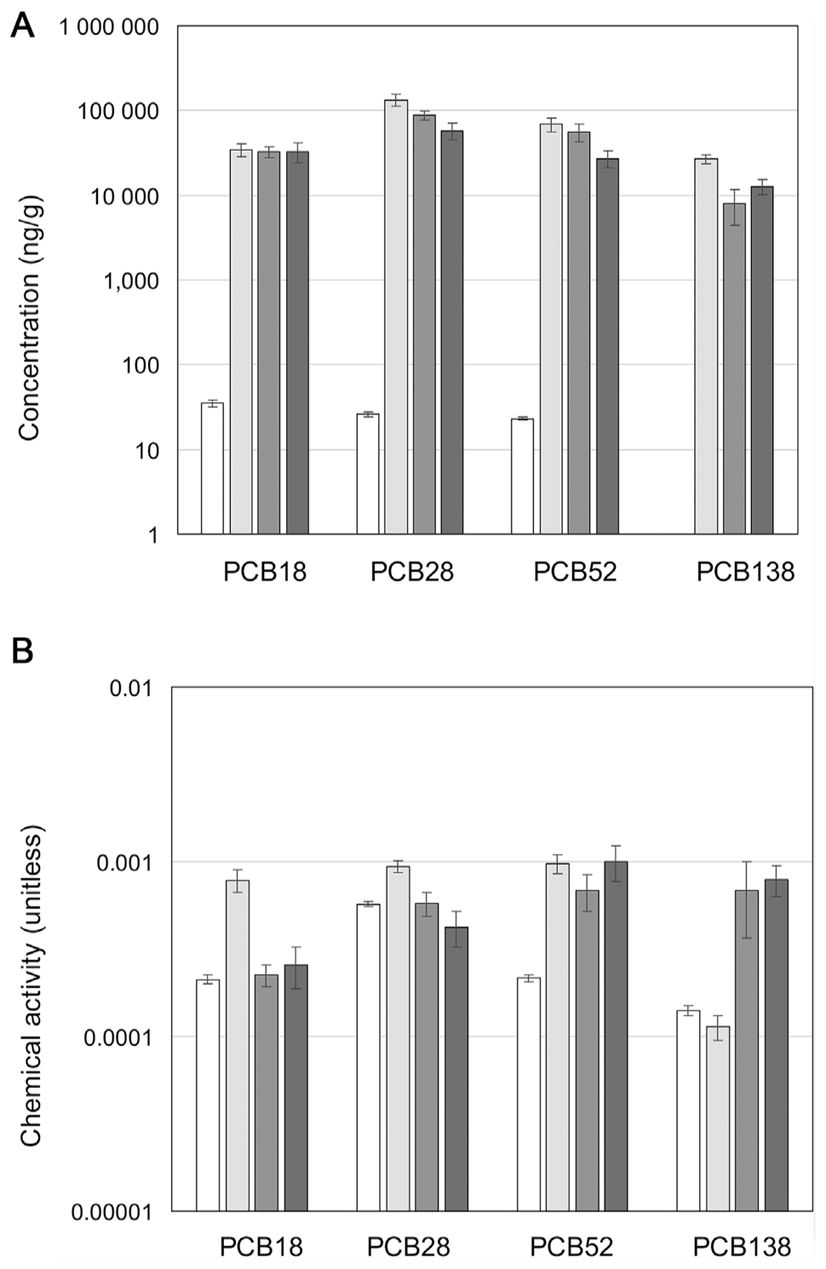
(**A**) Measured concentrations of several polychlorinated biphenyl congeners in water (white bars), sediments (light gray bars), and lipids of the polychaete (*Nereis virens*) (gray bars) and the mussel (*Mytilus edulis*) (dark gray bars) from the New Bedford Harbor Superfund site (New Bedford, MA, USA). (**B**) Chemical activities determined from concentrations in low-density polyethylene passive sampler, deployed in the water (white bars) and sediments (light gray bars), and concentrations in lipids of the polychaete (*N. virens*; gray bars) and the mussel (*M. edulis*; dark gray bars). Error bars represent standard errors of the mean. Original data are from Friedman et al. (2009) and Burgess et al. (2013). PCB = polychlorinated biphenyl.

To illustrate the application of chemical activity for investigating chemical exposure pathways, we calculated chemical activities from measured concentrations of several PCB congeners in sediments, water column, water column mussels (*Mytilus edulis*), benthic polychaetes (*Nereis virens*), and low-density polyethylene passive samplers deployed in the water column and sediments of the New Bedford Harbor Superfund site (New Bedford, MA, USA). Concentration data were taken from Friedman et al. (2009) and Burgess et al. (2013), and chemical activities were calculated according to Equations 6 and 11 using subcooled liquid aqueous solubility values for PCB congeners 18, 28, 52, and 138 obtained from Burkhard et al. (1985) and Shiu and Mackay (1986), and *K*_PW_ values reported in Grant et al. (2016) and Booij et al. (2016) (Figure 3). Concentrations of PCB 138 in bulk water samples were below the detection limit but were detected in the passive samplers deployed in the water phase. The concentrations of the PCB congeners in water, sediments, and mussels are difficult to compare and interpret because they are different quantities expressed in different units. For example, a simple comparison of the concentrations of PCBs 18 and 38 in water and sediments does not reveal the net sediment-to-water diffusive flux. However, chemical activity measurements using passive samplers show that chemical activities of PCBs 18 and 28 in sediment are greater than those in water, indicating net passive (diffusive) transport of these PCB congeners from sediment to water.

Another example is the apparent similarity in the concentration values of PCBs 18 and 28 in sediments, polychaetes, and mussels, which might be interpreted as sediments being the main source of these PCB congeners in the polychaetes and mussels. However, the chemical activity measurements using passive samplers indicate that this is not the case. Chemical activities of PCBs 18 and 28 in water, polychaetes, and mussels are equal, indicating that the chemical activities of PCBs 18 and 28 in mussels and polychaetes respond to and reflect the chemical activities in the water, despite the higher chemical activity in the sediment compared with that in the water. This behavior can be expected given that these relatively water-soluble PCB congeners are mainly absorbed via the water rather than the diet. In contrast, the chemical activity of PCB 52 in the sediments and organisms is similar to and greater than that in the water, suggesting that sediments are the primary source of exposure. Finally, the chemical activities of PCB 138 in polychaetes and mussels are greater than those in the water and sediment. This may indicate the role of dietary uptake and subsequent biomagnification. Biomagnification causes an increase in chemical activity in the organism over that in the water or sediment to which the organism is exposed (Connolly and Pedersen 1988; Gobas et al. 1999).

### Application of chemical activity to hazard assessment

The relationship between chemical activity and toxicity has been investigated by data compilation and analysis (Freidig et al. 1999; Mackay and Arnot 2011; Thomas et al. 2015) and by conducting experiments that involve passive dosing (Reichenberg and Mayer 2006; Mayer and Holmstrup 2008; Smith et al. 2013). In passive dosing experiments, a biocompatible polymer containing a test substance is included in the toxicity test as a donor of the test substance to control the chemical activity in the test in the form of the freely dissolved concentration of the chemical in the water (i.e., *C*_free_; Mayer and Holmstrup 2008; Smith et al. 2013). By administering the chemical through equilibrium partitioning, the chemical activity in the test system can be controlled. The studies revealed that there is a chemical activity range of 0.01 to 1 that is associated with narcosis or baseline toxicity. The empirical studies confirmed earlier estimates of the chemical activity range for baseline toxicity by Reichenberg and Mayer (2006) and Mackay and Arnot (2011) based on literature data compilations. Also, passive dosing experiments showed that substances with a high melting point (and hence a low fugacity ratio expressed by Equation 5) cannot achieve the relatively high chemical activities of 0.01 to 0.1 and do not exhibit narcosis-related effects in aquatic and terrestrial organisms (Mayer and Holmstrup 2008; Smith et al. 2010). This melting point cutoff was also suggested by Abernethy and Mackay (1987) to explain the apparent toxicity cutoff of chemicals with low aqueous solubilities and by Lipnick et al. (1987) to explain observations in the classic Overton (1896) study where anthracene and phenanthrene, which are isomers with similar partitioning properties, display different adverse outcomes—that is, phenanthrene, with a melting point of 99.5 °C, produces narcosis, whereas anthracene, with a melting point of 217.5 °C, does not—as further demonstrated in experiments by Mayer and Reichenberg (2006).

Passive dosing experiments also indicated a relatively narrow range of sensitivities to PAHs among the test organisms (Mayer and Reichenberg 2006; Smith et al. 2010, 2013; Rojo-Nieto et al. 2012; Seiler et al. 2014; Vergauwen et al. 2015; Butler et al. 2016). Furthermore, the studies showed that the baseline toxicity of PAH mixtures is closely linked to the sum of chemical activities of the PAHs in the PAH mixtures (Schmidt et al. 2013) and that PAHs that are unable to cause baseline toxicity individually because of a high melting point can contribute baseline toxicity in the presence of other PAHs in mixtures (Smith et al. 2013). These studies support the view that baseline toxicity of mixtures of chemicals can be expressed by the sum of chemical activities and that a sum of chemical activities greater than 0.01 is related to baseline toxicity in aquatic organisms. For modes of toxic action other than narcosis, the relationship between chemical activity and the toxic response is unclear. However, it is expected that chemicals exert modes of toxic action other than narcosis (or baseline toxicity) at chemical activities below 0.01. Effects occurring at chemical activities below 0.01 indicate excess toxicity (i.e., toxicity greater than baseline toxicity). The lower the chemical activity in the organism required to cause a harmful effect, the greater the excess toxicity.

**FIGURE 4:**
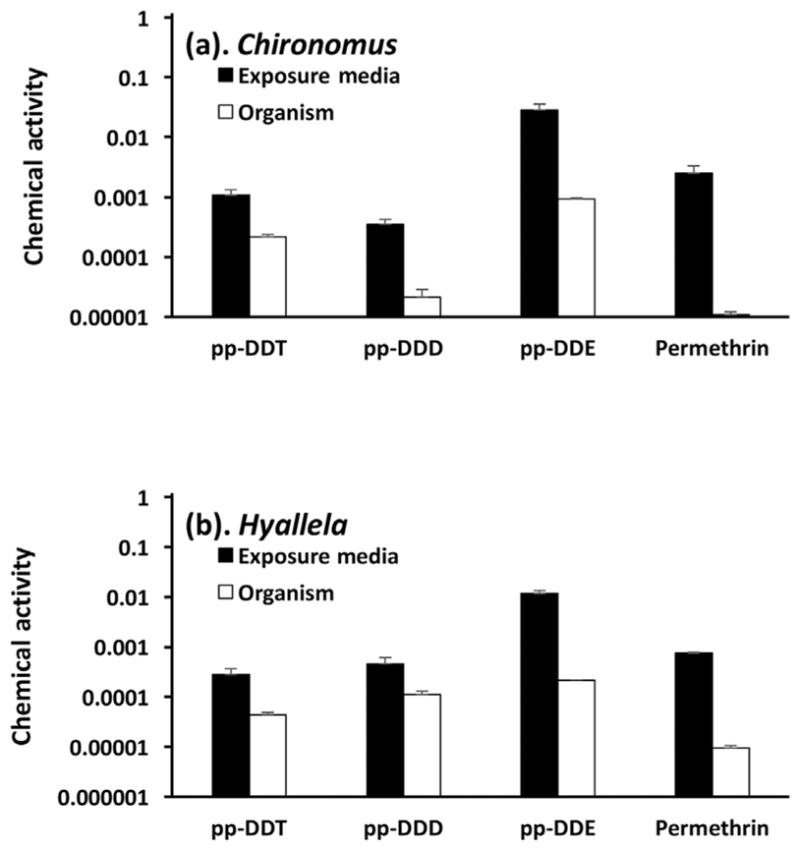
Reported 10-d 50% effect concentrations and 50% lethal concentrations of several pesticides in the exposure medium and the lipids of the aquatic invertebrate species *Chironomus dilutus* and *Hyalella azteca* expressed in terms of chemical activity. DDE = dichlorodiphenyldichloroethylene; DDD = dichlorodiphenyldichlorethane; DDT = dichlorodiphenyltrichloroethane.

Chemical activity may therefore be a useful diagnostic tool for elucidating a chemical’s potency for adverse effects or inherent toxicity. For example, Ding et al. (2012a, 2012b) reported the toxicity of several pesticides in *Chironomus dilutus* and *Hyalella azteca* in terms of the concentration of the pesticide in the polymer used for passive sampling (derived by equilibrating aqueous test media with fibers coated with polydimethylsiloxane) and the concentration of the pesticide in the lipids of the exposed test organisms (obtained by tissue and lipid analysis of test organisms collected at the end of the tests). We converted the concentrations in the passive sampling polymer into an external chemical activity and the concentrations in the lipids into an internal chemical activity (Figure 4) using the Activity Calculator (Gobas et al. 2015a) as described in the Supplemental Data. Figure 4 shows that toxicity was exerted at internal chemical activities in the organism that are far below the range of chemical activities associated with baseline toxicity. This indicates that these pesticides exhibit excess toxicity reflecting a specific mode of action different from narcosis. Furthermore, the chemical activity causing effects in both test species was lower for permethrin than for dichlorodiphenyltrichloroethane (DDT), dichlorodiphenyldichloroethylene (DDE), and dichlorodiphenyldichlorethane (DDD). This indicates that permethrin has a greater inherent toxicity than DDT, DDE, and DDD. Figure 4 also shows that the external activities of the chemical (derived from the concentrations in the polymer) were greater than the internal activities (derived from the concentrations in the lipids of the test organisms). This indicates that the test chemicals in the test organisms were not in thermodynamic equilibrium with the test chemicals in the exposure medium. This may have been attributable to an insufficient exposure duration or to biotransformation that reduces the chemical activity in the organism below that in the exposure medium. The chemical activity analysis of the experimental data illustrates the opportunities for measuring the toxicological potency of chemicals in terms of chemical activities. It also highlights the challenges of relating toxicity to external chemical activities in cases of insufficient exposure duration or significant biotransformation.

### Application of chemical activity to risk assessment of single chemicals

**FIGURE 5:**
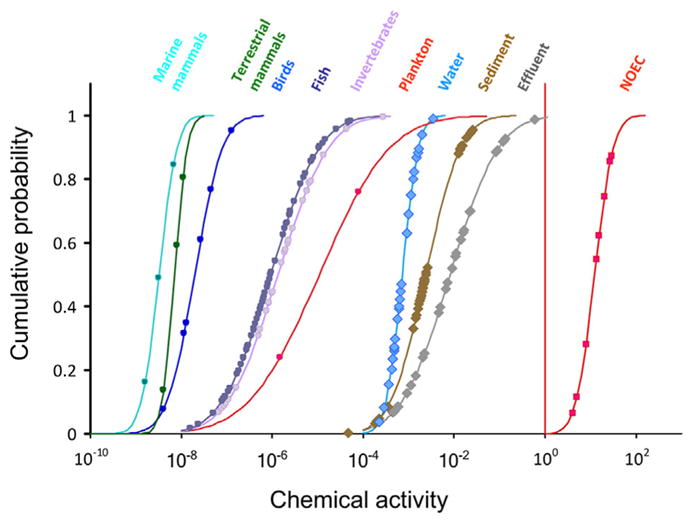
Cumulative probability distributions of chemical activities (unitless) of decamethylcyclopentasiloxane (D5) in effluents of D5 production units, ambient water, sediment, plankton, invertebrates, fish, birds, and terrestrial mammals from locations in northern Europe and the United States in relation to the maximum environmentally feasible chemical activity of D5 of 1 (red straight line) and apparent (but unrealistic) chemical activities of D5 greater than 1 corresponding to reported no-observed effects in toxicity assays. Markers depict chemical activities in individual studies. Original data are from Gobas et al. (2015b). NOEC = no-observed-effect concentration.

The chemical activity approach has been used as a method for conducting environmental risk assessments of several high-production commercial chemicals including decamethylcyclopentasiloxane (D5; Gobas et al. 2015b), octamethylcyclopentasiloxane (D4; Fairbrother and Woodburn 2016), diethylhexylphthalate ester (DEHP), and the DEHP metabolite mono-ethylhexylphthalate ester (MEHP; Gobas et al. 2016). In these risk assessments, exposure and toxicity data from many different sources were expressed in terms of chemical activities. The chemical activity–based risk assessment for D5 (Figure 5) illustrates that reported concentrations of D5 in various environmental media are thermodynamically feasible and do not indicate gross sampling errors (e.g., sample contamination, unit errors). In contrast, all reported NOECs for D5 correspond to chemical activities greater than 1 (Figure 5). This indicates that the reported NOECs were determined at concentrations above the solubility or the sorptive capacity of the dosing medium in the toxicity tests. The exposure medium in these tests likely included undissolved or neat D5 liquid, which normally does not occur in the environment. The toxicity test results may therefore not be ecologically relevant and informative for inclusion in an environmental risk assessment. The chemical activity–based risk assessment further illustrates that chemical activities of D5 in biota are in general much lower than those in water and sediment and appear to decrease with increasing trophic position, suggesting biotransformation of D5, trophic dilution, and a lack of biomagnification. Chemical activities of D5 in biota are also below the range of chemical activities associated with baseline toxicity (i.e., 0.01–1), which, in the absence of toxicity data, can be used as an initial hazard screening benchmark.

**FIGURE 6:**
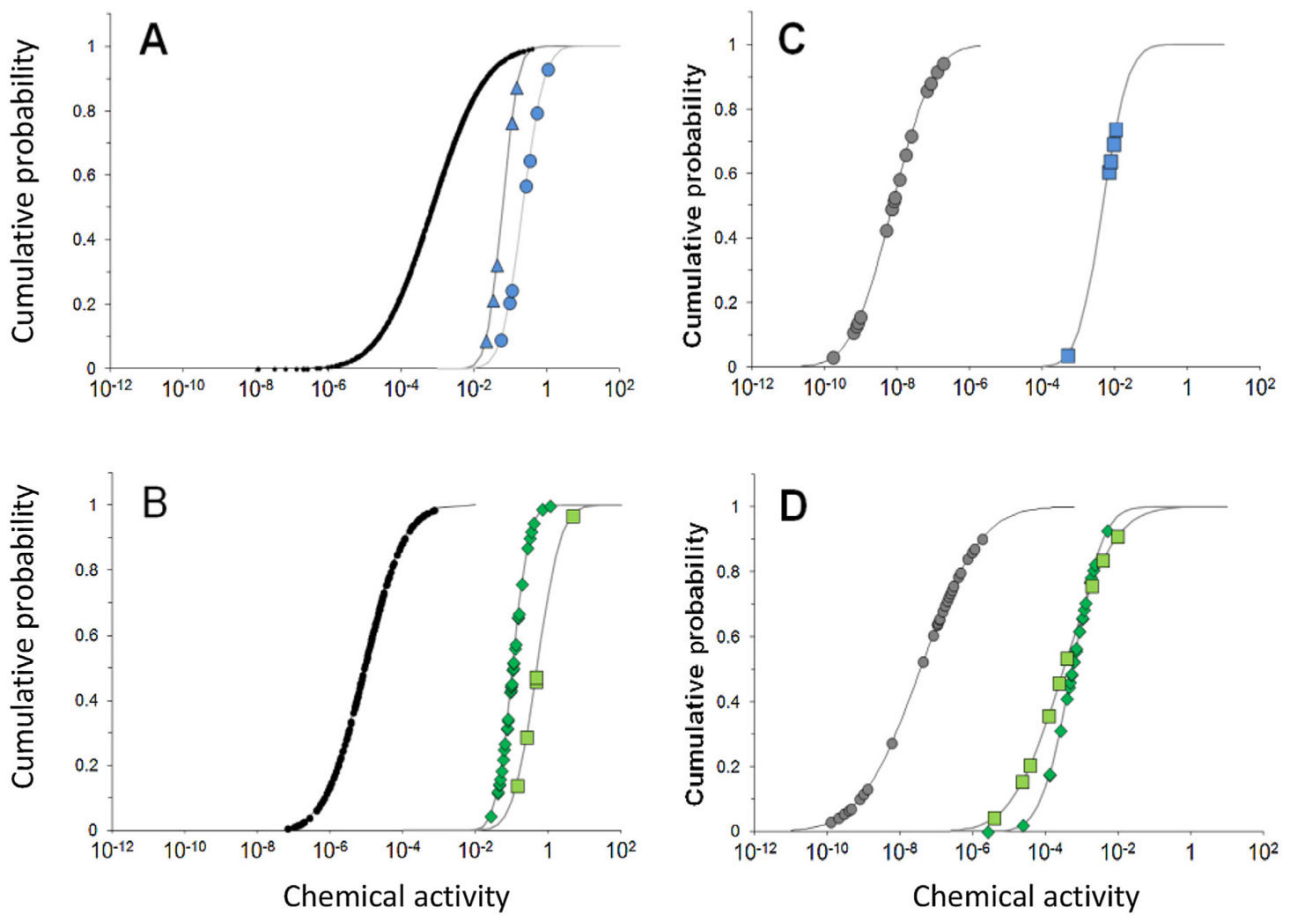
Cumulative probability distributions of chemical activities (unitless) of di-ethylhexylphthalate ester (DEHP) in abiotic (**A**) and biotic (**B**) media and of mono-ethylhexylphthalate ester (MEHP) in abiotic (**C**) and biotic (**D**) media in relation to the range of chemical activities associated with biological responses in in vivo and in vitro tests. (**A**) Chemical activities of DEHP corresponding to the exposure concentration of DEHP in abiotic media (black circles, *n* = 934 studies, circles appear as line), no-observed-effect concentrations of DEHP in in vivo tests (blue circles, *n* = 7 studies), and lowest-observed-adverse-effect levels of DEHP in in vivo tests (blue triangles, *n* = 6 studies). (**B**) Chemical activities of DEHP corresponding to concentration of DEHP in biota (gray circles, *n* = 197 studies), median effective concentrations (EC50s) or median inhibitory concentrations (IC50s) in in vitro tests (green squares, *n* = 5 studies), and Toxcast median active concentration (AC50s) in positive in vitro tests (green diamonds, *n* = 40 studies). (**C**) Chemical activities of MEHP corresponding to exposure concentration of MEHP in abiotic media (gray circles, *n* = 17 studies); EC50s or median lethal concentrations of MEHP in daphnia and fish (blue squares, *n* = 5 studies). (**D**) Chemical activities of MEHP corresponding to concentration of MEHP in biota (gray circles, *n* = 26 studies), EC50s or IC50s of MEHP in in vitro tests (green squares, *n* = 9 studies), and Toxcast AC50s of MEHP in positive in vitro tests (green diamonds, *n* = 26 studies). See Gobas et al. (2016) for further detail. ToxCast is the US Environmental Protection Agency’s Toxicity Forecaster.

The chemical activity–based risk assessment for DEHP and MEHP (Gobas et al. 2016) illustrates the application of the chemical activity approach to vast amounts of exposure and toxicity data including high-throughput in vitro toxicity data from the USEPA’s Toxicity Forecaster (ToxCast); data from in vitro bioassays for cytotoxicity, estrogenic activity, androgenic activity, anti–thyroid hormone activity, and receptor binding assays; and data from in vivo toxicity studies (Figure 6). The chemical activity analysis showed that biological responses in both in vivo and in vitro tests occur at chemical activities between 0.01 and 1 for DEHP and between approximately 10^−6^ and 10^−4^ for MEHP, indicating that DEHP is likely a baseline toxicant in toxicity experiments and that MEHP has a greater potency than DEHP. The similarity in chemical activities associated with biological effects in in vitro and in vivo tests for both DEHP and MEHP suggests that the chemical activity approach may be useful in the in vitro to in vivo extrapolation of biological effects. The mean chemical activity of DEHP in biological samples was on average approximately 100-fold lower than that in abiotic samples, indicating biotransformation of DEHP in biota, which has been confirmed in independent experiments (Barron et al. 1995). Chemical activities of both DEHP and MEHP in biota samples were less than those that produced biological activity in in vitro bioassays without exception. A small fraction of chemical activities of DEHP in abiotic environmental samples (i.e., 4–8%) and none (0%) for MEHP were found to be within the range of chemical activities associated with LOECs and NOECs in in vivo tests.

**FIGURE 7:**
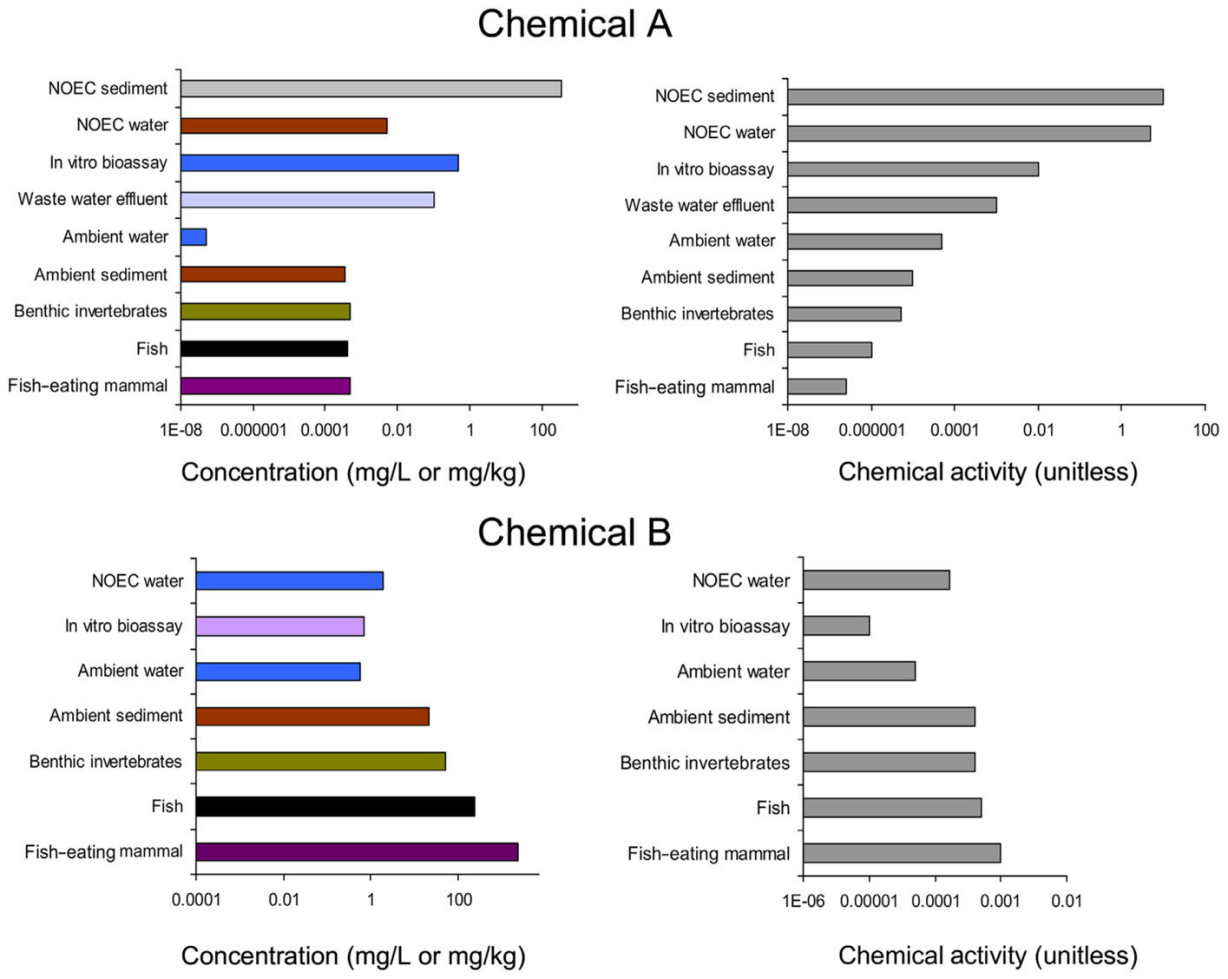
Concentrations (left) and chemical activities (right) of chemicals A and B in illustrative environmental scenarios evaluated for risk. NOEC = no-observed-effect concentration.

The examples of the application of chemical activity for risk assessment for D4, D5, DEHP, and MEHP illustrate some key features of a chemical activity–based risk assessment, namely 1) consideration of a wider array of data than is usually possible when using a concentration-based approach, 2) evaluation of empirical exposure data to confirm thermodynamic plausibility, 3) rudimentary risk assessment (for narcosis) in the absence of reliable toxicity information, 4) improved characterization of uncertainty in hazard and exposure data, and 5) identifying erroneous toxicological data. Boxes 1 and 2 provide additional illustrative examples of chemical activity–based risk evaluations of chemical substances. The boxes focus on differences between concentration and activity-based risk evaluations and illustrate that chemical activity–based analyses can achieve insights into the behavior, effects, and risks of chemicals that concentration-based analyses often cannot without further analysis. Box 1 is an example where risk may be overestimated in a concentration-based risk analysis (false positive), whereas Box 2 is an example where risk may be underestimated in a concentration-based risk analysis (false negative). The illustrative environmental scenarios are described in Tables 1 and 2. The concentration and chemical activity–based risk analyses are graphically illustrated in Figure 7.

**CONCENTRATION VS. CHEMICAL ACTIVITY BASED RISK EVALUATION**

Chemical A (log *K*_OW_ = 7, molecular weight is 400 g/mol, melting point is 0 °C) is an example of a very hydrophobic chemical that is liquid at 25 °C. Toxicity studies indicate a NOEC in a sediment toxicity study of a 1000 mg/kg and a NOEC of 0.005 mg/L in a chronic fish toxicity test. Table 1 and Figure 7 tabulate and illustrate the available concentration data for chemical A for the risk analysis. Calculation methods are detailed in the Supplemental Data.

A concentration data analysis (Figure 7 and Table 1) of the available data may conclude the following:

1. Chemical A is a highly toxic chemical because the NOEC of 0.005 mg/L in a chronic fish toxicity test is below the 0.1 mg/L criterion value for inherent toxicity in Canada or the 0.01 mg/L criterion under REACH.
2. Chemical A may be causing harm in the environment because the concentration of chemical A in wastewater effluents is 0.1 mg/L and above the NOEC of 0.005 mg/L.
3. Chemical A is a very bioaccumulative substance because the apparent bioaccumulation factor in fish is 8000 L/kg and above the criterion level of 5000 L/kg for a highly bioaccumulative substance and the biomagnification factor for fish-eating mammals is 1.25.

The chemical activity analysis would conclude the following:

1. Apparent NOECs have little relevance for the assessment of the toxicity of chemical A or for environmental risk assessment because chemical activities associated with the NOECs exceed 1, which indicates that exposure concentrations in the toxicity tests were above the aqueous solubility of chemical A (for the chronic fish toxicity study) and above the sorptive capacity of sediments for chemical A (for the sediment toxicity test). Furthermore, the fact that baseline toxicity of chemical A was not observed in in vivo studies indicates that concentrations of chemical A in the test organisms did not reach the concentrations in the organisms that are high enough to cause baseline toxicity. This may occur to a chemical that is biotransformed in the organism.
2. Chemical A appears to behave as a baseline toxicant in the in vitro bioassay and does not exhibit excess toxicity in the bioassay because the chemical activity associated with biological activity in in vitro bioassays is 0.01, equal to that expected from baseline toxicants.
3. Chemical A is not expected to cause harm to the environment in this scenario because the chemical activities of chemical A in all ambient media are less than the chemical activity in the in vitro bioassay and the unrealistic chemical activities associated with the NOECs in the 2 toxicity tests.
4. Chemical A does not show evidence of bioaccumulation in a thermodynamic sense because the chemical activity in the fish is lower than the chemical activity of chemical A in the water and 5 times lower than that in benthic invertebrates. Also, the chemical activity in the mammal is 4-fold less than that in its prey (fish).

### Application of chemical activity to risk assessment of multiple chemicals

**FIGURE 8:**
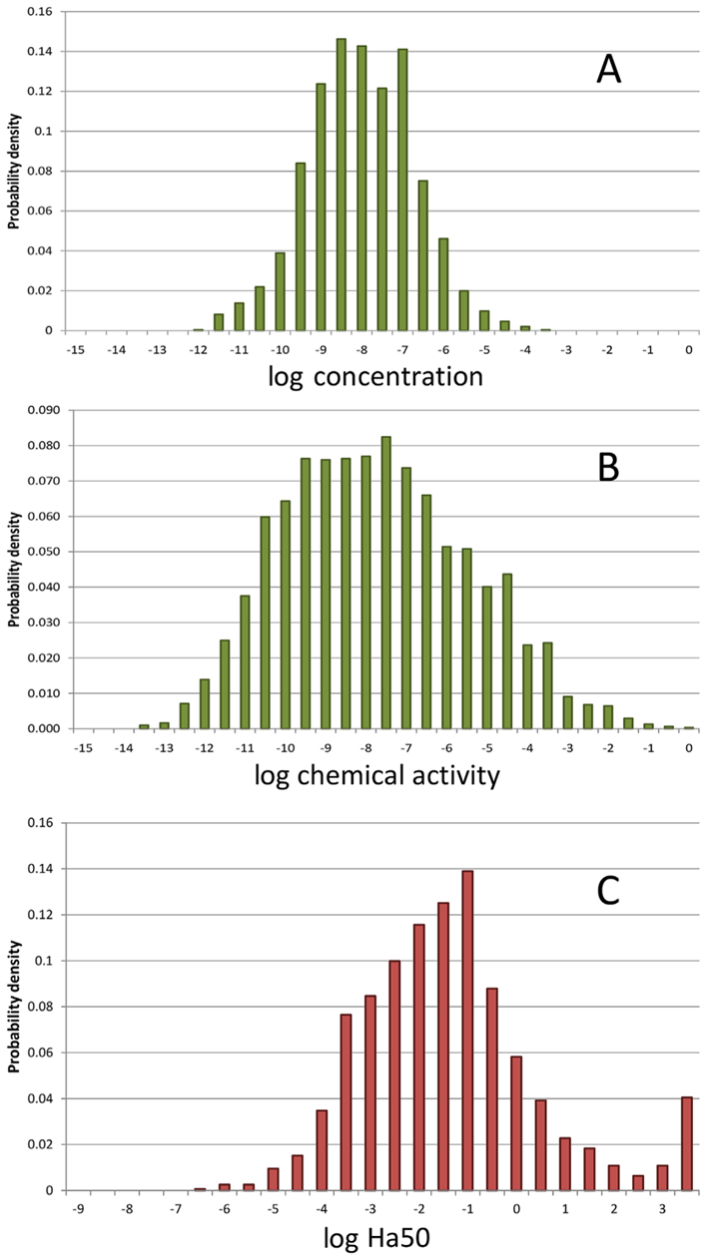
Distributions of (**A**) calculated concentrations of more than 3000 Registration, Evaluation, Authorisation and Restriction of Chemicals–registered substances in European Union regional water; (**B**) chemical activities corresponding to the concentrations depicted in (**A**); and (**C**) critical effect limits of the same substances expressed in terms of chemical activities. Ha50 = critical effect limit.

The chemical activity approach may also be useful in assessing the combined toxicity from mixtures of chemical substances. In recent studies for the European Commission (Seventh Framework Program, SOLUTIONS project) and the European Chemical Industry Council (Long-Range Research Institute project Time-Integrative Passive Sampling Combined with Toxicity Profiling), the narcotic toxic pressure of chemicals in natural freshwaters was both measured and calculated (Hamers et al. unpublished data; Van de Meent et al., unpublished data). Based on European Union–wide uses of Registration, Evaluation, Authorisation and Restriction of Chemicals (REACH)–registered chemicals, the authors 1) estimated European Union–wide release rates of more than 3000 monoconstituent organic substances into air, water, and soil; 2) calculated the expected concentrations of these substances in regional European Union freshwaters following REACH directives (Figure 8A); 3) deduced the distribution of expected chemical activities of the combined sum of REACH-registered substances in European Union waters (Figure 8B); and 4) derived critical effect limits (Ha50) of the same substances expressed in terms of chemical activities (Figure 8C).

**CONCENTRATION VS. CHEMICAL ACTIVITY BASED RISK EVALUATION**

Chemical B (log *K*_OW_ = 3, molecular weight is 200 g/mol, melting point is 0 °C) is an example of a chemical that is more water-soluble (aqueous solubility= 12 g/L) than chemical A. Concentrations of chemical B in various media in an illustrative environment are listed in Table 2. The NOEC of chemical B in a chronic fish toxicity test is 2 mg/L. A NOEC for benthic invertebrates is not available. Table 2 and Figure 7 tabulate and illustrate the available concentration data for chemical B. Calculation methods are detailed in the Supplemental Data.

A concentration-based analysis (Figure 7) may conclude the following:

1. Chemical B is not of toxicological concern because the NOEC is greater than the 0.1 mg/L criterion value for inherent toxicity in Canada or the 0.01 mg/L criterion under REACH.
2. chemical B is not causing harm in the environment because the concentration of chemical B in ambient water is 0.6 mg/L and lower than the NOEC of 2 mg/L. The concentration of chemical B in the sediment cannot be interpreted in terms of harm because of the lack of a NOEC for benthic invertebrates.
3. The analysis may conclude that chemical B is not bioaccumulative because the fish/water bioaccumulation factor of chemical B is 330 L/kg and below criteria levels of 2000 or 5000 used in regulations.

A chemical activity analysis (Figure 7) would conclude the following:

1. Chemical B is a substance of toxicological concern because the chemical activities of chemical B corresponding to the NOEC and the in vitro toxicity bioassay are 1.7 × 10^−4^ and 10^−5^, respectively, and below the chemical activity of 0.01associated with baseline toxicity. Hence, chemical B exhibits excess toxicity beyond baseline toxicity.
2. Chemical B has the potential to cause harm because the chemical activities of chemical B in sediments, invertebrates, fish, and mammals exceed the chemical activities associated with the NOEC in the chronic fish toxicity study and biological activity in the in vitro bioassay.
3. Chemical B bioaccumulates in a thermodynamic sense because the chemical activity of chemical B in the fish-eating mammal is greater than that in its prey.

**FIGURE 9:**
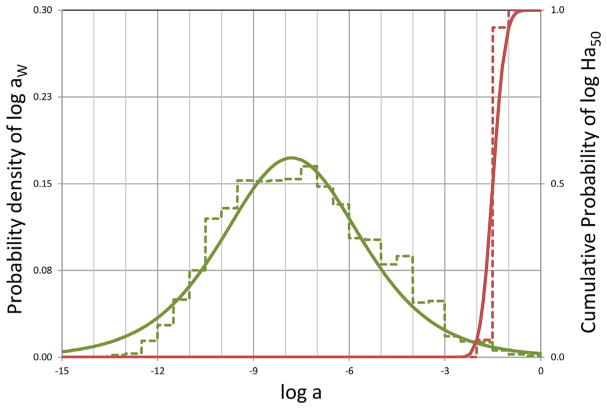
Gaussian probability density functions (solid line) and histograms (dashed line) for the logarithm of the combined chemical activities of more than 3000 chemicals registered under Registration, Evaluation, Authorisation and Restriction of Chemicals in regional European Union freshwater (green lines) and critical effect limits expressed in chemical activity (red lines), illustrating the narcotic toxic pressure in regional European Union freshwater, as obtained by Van de Meent et al. (unpublished data) as the overlap of the distributions of chemical activities in water (green line, left) and the distribution of critical effect limits (red lines, right). Note that the chemical activities of the more than 3000 chemicals in water range from approximately 10^−12^ to 10^−3^ and that the critical effect limit of Ha50 = 0.01 is exceeded only in a few instances. *a* = chemical activity; Ha50 = critical effect limit.

The authors then computed the narcotic toxic pressure using the calculation procedure described originally by Van Straalen (2002), Aldenberg et al. (2002), and Traas et al. (2002), who defined toxic pressure as the probability that critical effect concentrations for one or more aquatic organisms are exceeded by exposure concentrations of one or more chemical substances in a water system. Narcotic toxic pressure in regional European Union freshwater was derived from the overlap of the distribution of chemical activities of more than 3000 substances in water (*a_w_*), with the distribution of critical effect limits (Ha50), calculated from LC50s and EC50s. The distribution of the logarithm of the critical effect limits (log Ha50) was assumed to be normal, with a mean of −2 (i.e., a corresponding Ha50 of 0.01) and a standard deviation of 1 log unit (Van de Meent et al., unpublished data), as shown in Figure 9.

From these 2 distribution functions (Figure 9), it was determined that the probability of the combined chemical activity of the more than 3000 REACH substances in water exceeding 0.01 was approximately 1%. This means that any (randomly chosen) aquatic species in regional European Union freshwater has a probability of 1% to be exposed to chemical activity in water in excess of the critical limit of 0.01. Interestingly, Hamers et al. (unpublished data) found similar results via field monitoring of waters in The Netherlands using extensive passive sampling and toxicity profiling. It should be stressed that the chemical activity in biota is not necessarily the same as that in the waters to which the organisms are exposed. Hence, a high toxic pressure in the water does not necessarily imply that toxic effects will occur. For example, chemicals may be metabolized, thereby reducing the chemical activity in an organism relative to that in the water in which the organism resides. The study also revealed that acute LC50 and EC50 values submitted in REACH registration dossiers often exceed the reported solubility limits of the substances. Chemical activities calculated from the LC50 and EC50 in excess of 1 may reflect the confounding physical effects of undissolved test material rather than characterizing the inherent toxicity of the dissolved substance. Figure 8C shows that more than 20% of the calculated log Ha50 exceeded the aqueous solubility of the tested substances by up to a factor of 1000.

### Application of chemical activity to support guideline development

Another potential application of the chemical activity approach is in environmental quality guideline development. In many cases, the methodology for guideline development varies between environmental media (e.g., water, sediment, soil, animal tissue). This approach tends to produce a patchwork of medium-specific guideline values that are often not internally consistent and sometimes fail to achieve the objective of consistently protecting environmental and human health (Arblaster et al. 2015). A chemical activity approach allows toxicity data to be evaluated for a safe chemical activity value that can be applied to all relevant environmental media irrespective of the route of intake by the organism. The same approach may also be suitable to guide the safe use of ingredients in consumer products. The laws of thermodynamics ensure that when chemical concentrations in environmental media are held at or below the safe activity value, environmental quality standards are achieved. This approach cannot be applied to chemicals that can biomagnify, and other approaches should be considered (Alava et al. 2012; Arblaster et al. 2015). However, the great majority of chemicals in commerce do not biomagnify, and the application of chemical activity may serve as a simple, pragmatic strategy to develop environmental quality criteria for chemicals in multiple environmental media of varying composition. The safe chemical activity can be converted into a concentration for a specific medium that is of interest or concern by multiplying the safe chemical activity by the solubility of the chemical in the medium.

To monitor for exceedance of thermodynamically based environmental quality criteria in the environment, various standard techniques can be used. Passive sampling techniques may provide a particularly simple and economically attractive method for monitoring if environmental quality objectives are met (Mayer et al. 2014) because the concentrations of chemicals in samplers can be related to chemical activities in a highly reproducible manner (Grant et al. 2016). The equilibrium partitioning approach (Di Toro et al. 1991) has been used to develop sediment benchmarks for a range of organic chemicals (US Environmental Protection Agency 2003; Booij et al. 2016). These benchmarks were based on the use of the freely dissolved concentration of the chemical in interstitial water in sediments (*C*_free_), which is a proxy for chemical activity, and expressing *C*_free_ in terms of an organic carbon–normalized sediment concentration that, if exceeded, may result in adverse effects to freshwater and marine benthic organisms. With the advent of proven passive sampling methods (Ghosh et al. 2014), *C*_free_ no longer needs to be modeled but instead can be reliably measured directly. The chemical activity approach can be used as a tool for comparing the presence of chemicals (in terms of their chemical activity) in various environmental matrices (e.g., organisms, interstitial waters) to the chemical activity deemed safe by the guideline.

## CONCLUSIONS

The chemical activity approach can make significant contributions toward achieving the goals expressed in the twenty-first-century vision of exposure and risk assessment. The chemical activity approach provides a method for interpreting large amounts of information on exposure and toxicity of chemicals obtained from various sources, characterizing uncertainty, sensing the environment for harmful chemicals, and expressing risks attributable to exposure to single chemicals and mixtures of chemicals. The examples discussed in the present article illustrate that the chemical activity approach provides practical methods for measuring, monitoring, and evaluating chemicals that enter the environment.

The chemical activity approach provides a well-established, theoretically sound framework for conducting exposure, hazard, and risk assessments. Expressed either in terms of chemical activities or in the related form of fugacities, lipid-normalized concentrations, or freely dissolved concentrations of the chemical in water, the chemical activity approach has proven to be useful in both research and decision-making contexts. The merit of applying the chemical activity approach is that thermodynamic limits to the behavior of chemicals in the environment (e.g., solubility, transport, temperature) are recognized and that data and information of various types can be included in the environmental risk assessment and management of chemicals. Further, chemical activity is a useful approach to express the toxicity of single and multiple chemicals resulting from baseline toxicity and to identify substances that exhibit excess toxicity relative to narcosis.

A primary limitation of the chemical activity approach is its unfamiliarity. The development of databases and activity calculators may help to overcome this challenge. Many databases and estimation programs for chemical property data are already available (e.g., Klamt 1995; US Environmental Protection Agency 2004; Pence and Williams 2010; Brown et al. 2012; Endo and Goss 2014; Archem 2017) and can be augmented or adjusted to be directly applicable to a chemical activity approach. Measured chemical properties like partition coefficients and solubilities need to be converted and expressed in terms of multimedia solubilities, activity coefficients, and sorptive capacities required for the chemical activity calculations. Methods for the conversion of chemical activities to environmentally and experimentally relevant concentrations are also available. Chemical activity calculators exist for water, sediment, soil, air, and biological media of varying compositions as well as for experimentally relevant media such as incubation media in in vitro bioassays (Gobas et al. 2015a). Also, methods exist to calculate the chemical activity in in vitro bioassays used in high-throughput toxicity testing (Armitage et al. 2014).

The chemical activity approach can support an adaptive environmental management strategy (Holling 1978) where toxicological data from high-throughput in vitro testing and in vivo studies in the laboratory and the field are compiled and expressed in terms of chemical activities and then evaluated and assessed to identify safe chemical activities values. The safe chemical activities may also guide the production of safe products where the product’s chemical constituents do not exceed the safe chemical activities found through toxicity testing. These safe activities can be expressed in terms of environmental quality criteria for various environmental media and then monitored in the environment using various techniques. Monitoring using passive sampling techniques may be particularly suited for this purpose because of its ability to reliably relate freely dissolved concentrations to chemical activities.

There are several research activities that can be proposed to further support and facilitate the application of chemical activity for chemical stewardship. They include further study of the relationship between the mode of action of chemicals and chemical activity. Identifying general relationships between chemical activity and biological response such as those observed for narcosis is particularly useful. However, in the absence of such general behaviors, knowledge of the chemical activity in an organism or that in the exposure medium associated with the detected biological response(s) is sufficient to apply the chemical activity approach for conducting risk assessments. Chemical activity may also be a useful dose metric for linking molecular, cellular, and organismal events associated with adverse outcome pathways. Whereas chemical activities in the range of 0.01 to 1 are closely related to molecular initiating events associated with narcosis, there may be other ranges of activities associated with molecular initiating events critical to adverse outcome pathways other than narcosis that are not currently fully understood. Chemical activities may be useful as a tool to expand the understanding of molecular initiating events and adverse outcome pathways. Research into the solubilities and sorptive capacities of chemicals in relevant environmental media such as human, animal, and plant tissues, sediments, soil, water, and passive sampling media will also help to advance the application of the chemical activity approach. Finally, the error and uncertainty associated with the conversion between concentrations and chemical activities need to be fully explored, minimized, and accounted for in the application of the chemical activity approach to exposure and risk assessment.

## Supplementary Material

supplement

## Figures and Tables

**TABLE 1:** Concentrations, solubilities, and chemical activities of chemical A in an illustrative environmental scenario

Available data	Concentration (mg/L or mg/kg)	Solubility (mg/L or mg/kg)	Chemical activity (unitless)
NOEC in sediment toxicity	350	35	10
NOEC in aquatic toxicity	0.005	0.001	5
In vitro bioassay	0.50	50	0.010
Wastewater effluent	0.1	100	0.001
Ambient water	5.10^−8^	0.001	5.0 × 10^−5^
Ambient sediment	3.5 × 1.0^−4^	35	1.0 × 10^−5^
Benthic invertebrates	5 × 10^−4^	100	5.0 × 10^−6^
Fish	4 × 10^−4^	400	1.0 × 10^−6^
Fish-eating mammal	5 × 10^−4^	2000	2.5 × 10^−7^

NOEC = no-observed-effect concentration.

**TABLE 2:** Concentrations, solubilities, and chemical activities of chemical B in an illustrative environmental scenario

Available data	Concentration (mg/L or mg/kg)	Solubility (g/L or g/kg)	Chemical activity (× 10^−3^, unitless)
NOEC in aquatic toxicity test	2.0	12	0.17
In vitro bioassay	0.72	72	0.010
Wastewater effluent	1.0	132	0.0076
Ambient water	0.6	12	0.050
Ambient sediment	17	42	0.40
Benthic invertebrates	53	132	0.40
Fish	197	490	0.40
Fish-eating mammal	236	2360	1.0

OEC = no-observed-effect concentration.
