# Supplementary material for: A Chemical Activity Approach to Exposure and Risk Assessment of Chemicals
Source: Environ Toxicol Chem. Author manuscript; Available in PMC 2019 May 1. (PMC5994922; doi:10.1002/etc.4091)
Supplement: supplement [file NIHMS971791-supplement.pdf]

Supporting Information

A Chemical Activity Approach to Exposure and Risk Assessment of Chemicals

Frank A.P.C. Gobas†*, Philipp Mayer‡, Thomas F. Parkerton§, Robert M. Burgess||, Dik van de Meent#, Todd Gouin††

† School of Resource and Environmental Management, Simon Fraser University, Burnaby, British Columbia, Canada V5A 1S6

‡ DTU Environment, Department of Environmental Engineering, Technical University of Denmark

§ Toxicology & Environmental Science Division, ExxonMobil Biomedical Sciences Inc., Houston, Texas, USA

|| U.S. Environmental Protection Agency, ORD/NHEERL, Atlantic Ecology Division, , Narragansett, Rhode Island, USA

#Radboud University Nijmegen, Department of Environmental Science P.O. box 9010, 6500GL Nijmegen, The Netherlands

†† TG Environmental Research, Sharnbrook, Bedfordshire, United Kingdom

*Address correspondence to gobas@sfu.ca

Tables

Table S1: Summary of recent literature studies (2012 – present) where chemical activity gradients between media were characterized using passive sampling measurements.

Passive Sampler	Environmental Gradient Investigated	Location and Date	Contaminants	References
LDPE	Surface waters – overlying atmosphere	Newark Bay (New Jersey, USA) 2008 - 2009	Dioxins/furans	S1
PDMS	Water column – sediment interstitial water - biota	Stockholm Archipelago 2009-2010	PCBs	S2
LDPE	Water column (surface and bottom) – sediment interstitial water	Narragansett Bay (Rhode Island, USA) 2009	PBDEs	S3
LDPE	Water column – sediment interstitial water	Hailing Bay (South China) 2012	DDTs	S4
LDPE	Surface waters – overlying atmosphere	Tropical Atlantic Ocean 2009	PBDEs	S5
LDPE, POM, PDMS	Water column – sediment interstitial water	Pacific Coast (California, USA) 2011	PCBs and DDTs	S6
PDMS	Sediment interstitial water – biota	Lake Ången (Sweden) 2012	PCBs and HCBs	S7,S8
LDPE	Water column – sediment interstitial water - biota	Passaic River (New Jersey, USA) 2011 - 2012	PCBs, PAHs and dioxins/furans	S9
LDPE	Surface waters – overlying atmosphere	Lower Great Lakes 2011	Organochlorine pesticides	S10
LDPE	Surface waters – overlying atmosphere	Lake Erie and Lake Ontario 2011	PCBs	S11
Polyurethane/ Silicone rubber	Surface waters – overlying atmosphere	Aegean Sea, 2012	PAHs, PCBs, PBDEs and organochlorine pesticides	S12
PDMS	Water column – sediment interstitial water	Baltic Sea 2008	PAHs	S13
LDPE and PDMS	Water column – sediment interstitial water - biota	Lake Kernaalanjarvi (Southern Finland) 2010	PCBs	S14
LDPE	Surface waters – overlying atmosphere	Lake Superior 2011	PAHs and PBDEs	S15
LDPE	Surface waters – overlying atmosphere	Gulf of Mexico 2010-2011	PAHs and oxygenated PAHs	S16
LDPE	Surface waters – overlying atmosphere	South and southeast Brazil	Organochlorine pesticides	S17

		2012		

Supporting Information Chemical Activity Calculations in Toxicity Tests

Ding et al. [S18,S19] expressed 10 d toxicity tests for *Chironomous dilutes* and *Hyalella azteca* in terms of concentrations on 10 micron polydimethylsiloxane (PDMS) coated fibers that were equilibrated with test media samples collected from various treatments with different pesticide concentrations for up to 60 days. In addition, effects were also related to the lipid normalized tissue concentrations in exposed test organisms. To calculate chemical activities from the equilibrium concentrations of the test chemicals in PDMS and lipids, sub-cooled liquid aqueous solubilities ($S_{W,L}$), PDMS-water (K_{PDMS}) and octanol-water partition (K_{OW}) coefficients for the four substances were obtained from the literature. For DDT and related compounds, $S_{W,L}$ and K_{OW} were taken from the critical review by Shen and Wania [S20] and K_{PDMS} from the recent work by Eganhouse [S21]. For permethrin, experimental water solubility (6 µg/L) and log K_{ow} (6.5) measurements were obtained from EpiSuite [S22]. The physico-chemical property data apply to a temperature 25 °C which was close to the 23°C temperature used in toxicity experiments. Since permethrin is a solid at room temperature (melting point = 34 °C), equation 5 was applied to calculate the subcooled liquid solubility of permethrin in water state, i.e. 7.4 µg/L. The K_{PDMS} value for permethrin was selected from the critical review by [S23]. The Activity Calculator [S24] was used to convert concentrations in octanol and PDMS into chemical activities. The chemical properties used in the chemical activity calculations are listed in Tables S1-S3. The calculations assume that the activity coefficient of the test chemicals in octanol can be used to approximate the activity coefficient in the organism's lipids. To calculate the chemical activities of the test chemical in PDMS, the log K_{PDMS} was used instead of log K_{OW} in the

Activity calculator. The toxicity data expressed in terms of PDMS or lipid normalized concentrations are reproduced from [S18] and [S19] in Table S3. The calculated chemical activities are summarized in Table S3 and plotted in Figure 4 in the main text.

Table S1: Input parameters used for the chemical activity calculations of experimental data presented in Ding et al. [S18,S19].

Chemical Activity Calculations for PDMS

Chemical Name:	DDT	DDD	DDE	Permethrin
Environmental Temperature (^{o}C)	23	23	23	23
Standard Temperature (^{o}C)	25	25	25	25
Molecular Weight (g/mol)	354.49	320.034	318.02	391.28
log Kow	5.76	5.1	6.05	5.59
Aqueous Solubility at Standard temperature (mol.m^{-3})	4.20E-07	2.30E-06	7.89E-07	2.04E-08
Vapor Pressure at Standard temperature (Pa)	na	na	na	na
Melting Point (^{o}C)	0	0	0	0
Enthalpy of Solution for the liquid or subcooled liquid (kJ.mol^{-1})	0	0	0	0
Enthalpy of Vaporization for the liquid or subcooled liquid (kJ.mol^{-1})	0	0	0	0

Chemical Activity Calculations for Octanol

Chemical Name:	**DDT**	**DDD**	**DDE**	**Permethrin**
Environmental Temperature (^{o}C)	23	23	23	23
Standard Temperature (^{o}C)	25	25	25	25
Molecular Weight (g/mol)	354.49	320.034	318.02	391.28
log Kow	6.39	6.33	6.93	6.5
Aqueous Solubility at Standard temperature (mol.m^{-3})	4.20E-07	2.30E-06	7.89E-07	2.04E-08
Vapor Pressure at Standard temperature (Pa)	na	na	na	na
Melting Point (^{o}C)	0	0	0	0
Enthalpy of Solution for the liquid or subcooled liquid (kJ.mol^{-1})	0	0	0	0
Enthalpy of Vaporization for the liquid or subcooled liquid (kJ.mol^{-1})	0	0	0	0

Table S2. Summary of Test Substance Properties at 25 °C, including molecular weight, subcooled liquid solubility in water ($S_{W,L}$) and the logarithm of the partition coefficients of the chemical between octanol and water (K_{OW}) and between PDMS and water (K_{PDMS}).

Substance	CAS#	Molecular Weight (g/mol)	$S_{W,L}$ (µg/L)	Log K_{OW} ($L_{water}/L_{octanol}$)	Log K_{PDMS} (L_{water}/L_{pdms})
pp-DDT	50-29-3	354.49	149	6.39	5.76
pp-DDD	72-54-8	320.034	736	6.33	5.10
pp-DDE	72-54-9	318.02	251	6.93	6.05
Permethrin	52645-53-1	391.28	8	6.50	5.59

Table S3. Toxicity data expressed in terms of fiber and lipid normalized organism concentrations and corresponding chemical activities as calculated by the Activity Calculator Version 1.2*.

Substance	*Chironomus* 10 d EC_{50} (μg/ml_{PDMS})	*Hyalella* 10 d LC_{50} (μg/ml_{PDMS})	*Chironomus* 10 d EA_{50} (a)	*Hyalella* 10 d LA_{50} (a)	*Chironomus* 10 d ER_{50} (μg/g_{lipid})	*Hyalella* 10 d LR_{50} (μg/g_{lipid})	*Chironomus* 10 d EA_{50} (a)	*Hyalella* 10 d LA_{50} (a)
pp-DDT	92.5	24.2	1.07E-03	2.82E-04	79	16	2.16E-04	4.38E-05
pp-DDD	32.5	42.4	3.51E-04	4.58E-04	33	175	2.10E-05	1.11E-04
pp-DDE	7992	3285	2.84E-02	1.17E-02	1989	454	9.31E-04	2.13E-04
Permethrin	7.8	2.3	2.51E-03	7.41E-04	0.27	0.24	1.07E-05	9.51E-06

*For the calculations of the chemical activities in the Activity Calculator, we used na (not applicable) for the vapor pressure, 0°C for the melting point (because the aqueous solubility applies to the subcooled liquid and hence the calculations need to treat the chemical as a liquid), and environmental temperature of 23°C and a standard temperature of 25°C.

References

S1.Friedman CL, MG Cantwell, R Lohmann. 2012. Passive sampling provides evidence for Newark Bay as a source of polychlorinated dibenzo-p-dioxins and furans to the New York/New Jersey, USA, atmosphere. Environ Toxicol Chem 31(2):253-261.

S2.Jahnke A, P Mayer, MS McLachlan. 2012. Sensitive Equilibrium Sampling To Study Polychlorinated Biphenyl Disposition in Baltic Sea Sediment. Environ Sci Technol 46(18):10114-10122.

S3.Sacks VP, R Lohmann. 2012. Freely dissolved PBDEs in water and porewater of an urban estuary. Environ Pollut 162:287-293.

S4.Liu H-H, L Bao, K Zhang, et al. 2013. Novel Passive Sampling Device for Measuring Sediment Water Diffusion Fluxes of Hydrophobic Organic Chemicals. Environ Sci Technol 47(17):9866-9873.

S5.Lohmann R, J Klanova, P Kukucka, et al. 2013. Concentrations, Fluxes, and Residence Time of PBDEs Across the Tropical Atlantic Ocean. Environ Sci Technol 47(24):13967-13975.

S6.Fernandez LA, W Lao, K Maruva, et al. 2014. Calculating the Diffusive Flux of Persistent Organic Pollutants between Sediments and the Water Column on the Palos Verdes Shelf Superfund Site Using Polymeric Passive Samplers. Environ Sci Technol 48(7):3925-3934.

S7.Jahnke A, M MacLeod, H Wickström, P Mayer. 2014. Equilibrium Sampling to Determine the Thermodynamic Potential for Bioaccumulation of Persistent Organic Pollutants from Sediment. Environ Sci Technol 48(19):11352-11359.

S8. Jahnke A, P Mayer, MS. McLachlan, H Wickström, D Gilbert, M MacLeod. 2014. Silicone passive equilibrium samplers as ‘chemometers’ in eels and sediments of a Swedish lake. Environ. Sci.: Processes Impacts, 2014, 16, 464.

S9.Khairy MA, MP Weinstein, R Lohmann. 2014. Trophodynamic Behavior of Hydrophobic Organic Contaminants in the Aquatic Food Web of a Tidal River. Environ Sci Technol 48(21):12533-12542.

S10.Khairy M, D Muir, C Teixeira, et al. 2014. Spatial Trends, Sources, and Air Water Exchange of Organochlorine Pesticides in the Great Lakes Basin Using Low Density Polyethylene Passive Samplers. Environ Sci Technol 48(16):9315-9324.

S11.Khairy M, D Muir, C Teixeira, et al. 2015. Spatial Distribution, Air Water Fugacity Ratios and Source Apportionment of Polychlorinated Biphenyls in the Lower Great Lakes Basin. Environ Sci Technol 49(23):13787-13797.

S12.Lammel G, O Audy, A Besis, et al. 2015. Air and seawater pollution and air sea gas exchange of persistent toxic substances in the Aegean Sea: spatial trends of PAHs, PCBs, OCPs and PBDEs. Environ Sci Pollut R 22(15):11301-11313.

S13.Lang S-C, A Hursthouse, P Mayer, et al. 2015. Equilibrium passive sampling as a tool to study polycyclic aromatic hydrocarbons in Baltic Sea sediment pore-water systems. 2015. Mar Pollut Bull 101(1):296-303.

S14.Mäenpää K, MT Leppanen, K Figueiredo, et al. 2015. Fate of polychlorinated biphenyls in a contaminated lake ecosystem: Combining equilibrium passive sampling of sediment and water with total concentration measurements of biota. 2015. Environ Toxicol Chem 34(11):2463-2474.

S15.Ruge Z, D Muir, P Helm, et al. 2015. Concentrations, Trends and Air Water Exchange of PAHs and PBDEs Derived from Passive Samplers in Lake Superior in 2011. Environ Sci Technol 49(23):13777-13786.

S16.Tidwell LG, SE Allan, SG O’Connell, et al. 2015. Polycyclic Aromatic Hydrocarbon (PAH) and Oxygenated PAH (OPAH) Air Water Exchange during the Deepwater Horizon Oil Spill. Environ Sci Technol 49(1):141-149.

S17.Meire RO, M Khairy, AC Targino, et al. 2016. Use of passive samplers to detect organochlorine pesticides in air and water at wetland mountain region sites (S-SE Brazil). Chemosphere 144:2175-2182.

S18.Ding Y, PF Landrum, JY Amanda D Hartwood, MJ. Lydy, (2012). Use of solid phase microextraction to estimate toxicity: Relating fiber concentrations to toxicity—part I. *Environ. Toxicol. Chem.* 31 (9), 2159–2167.

S19.Ding Y, PF Landrum, JY Amanda D Hartwood, MJ. Lydy, (2012). Use of solid phase microextraction to estimate toxicity: Relating fiber concentrations to body residues—part II. *Environ. Toxicol. Chem.* 31 (9), 2168–2174.

S20.Shen, L, F Wania 2005). Compilation, Evaluation and Selection of Physical-Chemical Property Data for Organichlorine Pesticides, *J. Chem. Eng. Data* 50:742-768.

S21.Eganhouse, RP 2016. Determination of polydimethylsiloxane-water partition coefficents for ten 1-chloro[2,2,2-trichloro-1-(-4-chlorophenyl)ethyl]benzene-related compounds and twelve polychlorinated biphenyls using gas chromatography/mass spectroscopy*, J. Chrom. A* 1438:226-235.

S22.US EPA. [2012]. Estimation Programs Interface Suite™ for Microsoft® Windows, v 4.11. United States Environmental Protection Agency, Washington, DC, USA.

S23.DiFilippo, EL, RP Eganhouse (2010). Assessment of PDMS-water partition coefficients: Implications for passive environmental sampling of hydrophobic organic compounds: *Env. Sci Technol.* 44:6971-6925.

S24.Gobas FAPC, Otton SV, Tupper-Ring LF, Crawford MA. 2015. Chemical activity Calculator for Solid and Liquid Non-Polar Organic Chemicals in Environmental Media. 2015. Environmental Toxicology Research Group at Simon Fraser University. http://www.sfu.ca/rem/toxicology/our-models/activity-calculator.html.

Supporting Information for Inserts

In inserts A and B, chemical activities are derived from the concentration data by dividing the concentrations by the "solubilities" of chemical A and B in the media in which the chemical was reported (Table 1 and 2). The solubilities of chemical A and B in the various media are calculated using the chemical activity calculator [S24] using the following input parameters:

Ambient Water and NOEC-water:

Concentration of particulate matter in water: 0 kg/L

Concentration of lipid in water: 0 kg/L

Concentration of protein in water: 0 kg/L

Salinity: 0 mol/L

Waste Water Effluent:

Concentration of particulate matter in water: 0 kg/L

Concentration of lipid in water: 0.01 kg/L

Concentration of protein in water: 0 kg/L

Salinity: 0 mol/L

In-vitro Bioassay:

Concentration of particulate matter in water: 0 kg/L

Concentration of lipid in water: 0.005 kg/L

Concentration of protein in water: 0 kg/L

Salinity: 0 mol/L

Sediment:

Organic carbon content of sediment particles: 0.01 kg/kg

Invertebrates:

Lipid content: 0.01 kg/kg

Protein content: 0 kg/kg

Carbohydrate content: 0 kg/kg

Fish:

Lipid content: 0.04 kg/kg

Protein content: 0 kg/kg

Carbohydrate content: 0 kg/kg

Fish Eating Mammal:

Lipid content: 0.20 kg/kg

Protein content: 0 kg/kg

Carbohydrate content: 0 kg/kg

For simplicity, the temperature of all media was assumed to be 25°C. The default parameters for the densities are used. Densities of water, lipids, organic carbon, protein and sediment particulate matter are respectively, 1, 0.9, 1, 0.9 and 1.2 kg/L.
